# Genetic polymorphisms in C-reactive protein increase cancer susceptibility

**DOI:** 10.1038/srep17161

**Published:** 2016-02-25

**Authors:** Peiliang Geng, Rina Sa, Jianjun Li, Hongtao Li, Chen Liu, Yunmei Liao, Lisha Xiang, Ning Wang, Juanjuan Ou, Ganfeng Xie, Houjie Liang

**Affiliations:** 1 Department of Oncology and Southwest Cancer Center, Southwest Hospital Third Military Medical University, 29 Gaotanyan Main Street, Chongqing 400038, China

## Abstract

Elevated levels of C-reactive protein (*CRP*) partially induced by polymorphisms in the *CRP* gene have been associated with human cancer. The purpose of this study was to test the hypothesis that *CRP* gene polymorphisms (+942G>C, 1846C>T) modify inherited susceptibility to cancer. We systematically identified the publications addressing the association of *CRP* gene polymorphisms with cancer susceptibility. Studies that fulfilled all inclusion criteria were considered eligible in this meta-analysis. We analyzed a total of 8 case-control studies. Individuals with the CC genotype were found to have an almost 4 fold higher risk of cancer than those with the GG or GC and GG genotypes. A significant association was also indicated in subgroup of colorectal cancer. Meta-analysis of 1846C>T polymorphism showed increased cancer risk in relation to the 1846 TT genotype (TT vs. CC: OR = 1.15, 95% CI = 1.01–1.31; TT vs. CT + CC; OR = 1.17, 95% CI = 1.03–1.32). Similar results were suggested in Caucasian populations and colorectal cancer. These data suggest that both +942G>C and 1846C>T polymorphisms in the *CRP* gene may influence cancer susceptibility.

Inflammation characterized by release of reactive oxygen/nitrogen species, formation of new blood vessels, degradation of tissues, induction of proliferation and inhibition of apoptosis is a pathophysiologic process involved in oncogenesis via various pathways^1^. A significant correlation between inflammation and human cancer was first established almost 20 decade years ago^2^, and inflammatory reactions have received widespread attention in cancer community ever since. Multiple epidemiologic and experimental studies have presented evidence supporting a causative role of chronic inflammation in carcinogenesis of numerous cancers^3^,^4^,^5^. Inflammation is mediated by cytokines and understanding the relevance of pro-inflammatory cytokine pathways to cancer aetiology may gain deeper insights into the molecular mechanisms.

C-reactive protein (*CRP*), known as an inflammatory biomarker, is generated by liver in response to *IL*-*6* which in turn upregulates serum levels of *CRP*^6^. Higher levels have been associated with the onset and development of cancer^7^,^8^. The *CRP* gene located at chromosome 1q21–1q23 consists of two exons and spans 1.9 kb in length. To date, there have been 29 single nucleotide polymorphisms (SNPs) identified in the *CRP* gene (http://www.ncbi.nlm.nih.gov/SNP). A panel of SNPs is shown to regulate *CRP* levels in the blood^9^,^10^,^11^,^12^. Therefore, an ideal way to investigate the role of human *CRP* gene in cancer susceptibility is to estimate the impact of SNPs within the region on the malignant progression.

Recently, retrospective and prospective studies in diverse populations have examined the association between cancer susceptibility and *CRP* SNPs^13^,^14^,^15^, with two non-synonymous polymorphisms (+942G>C, dbSNP ID: rs1800947; 1846C>T, dbSNP ID: rs1205) most extensively studied. However, there is substantial discrepancy in the results most likely due to the relatively small sample size. The goal of this meta-analysis was to comprehensively examine the relationship between the two *CRP* SNPs and cancer susceptibility.

## Methods

### Publication search

The Web of Science, Embase and PubMed were searched exhaustively using search terminology ((polymorphism) OR (polymorphisms)) AND ((C-reactive protein) OR (*CRP*)) AND (cancer) and their synonyms (variants, carcinoma, tumor, neoplasm) to identify the publications reporting on *CRP* polymorphisms and cancer risk. The electronic search lasted eight months. Additional usable data were obtained by hand searching the bibliographies of genetic association studies on the subject in this analysis. We used no restrictions on the number of samples and language to minimize publication bias.

### Inclusion criteria and exclusion criteria

Studies were considered in this analysis if the following conditions were fulfilled: (1) a case-control study with cancer patients investigated; (2) the relationship between *CRP* polymorphisms and cancer risk was assessed; (3) genotype frequency of the same polymorphism must be available in at least four studies; (4) the study must be unique without any subsequent update. We excluded the studies where the controls were cancer patients and genotype data were unaccessible even after having contacted corresponding authors.

### Data extraction

For the studies included, two investigators collected the first author’s surname, publication year, study country, ethnicity, cancer type, number of genotyped cases and controls, source of controls, genotyping methods and genotype frequency. Ethnicity was categorized as East Asian or Caucasian. Samples from the USA were grouped into Caucasian ethnicity and those from China and Japan were considered as East Asian ethnicity. We counted the different cancer types and ethnic populations reported in the same article as separate studies that were appropriately classified into the category described above.

### Statistical methods

Cancer risk in relation to *CRP* polymorphisms was estimated by crude ORs and 95% CIs (OR, odds ratio; 95% CI, confidence interval). We calculated the pooled ORs using multiple genetic models (Table 1). Subgroup analyses by cancer type was performed for *CRP* SNP +942G>C, while for *CRP* SNP 1846C>T, data were stratified by ethnicity in addition to cancer type.

Heterogeneity across studies was evaluated by the Chi square-based Q-test, and a *P* value more than .10 indicated the effect size was homogeneous. We combined OR for the single studies using the Mantel-Haenszel method unless little heterogeneity was indicated, or else the DerSimonian and Laird method was used^16^,^17^. Hardy-Weinberg equilibrium (HWE) was examined by using the χ^2^ test in the control group of each study. Sensitivity analysis by sequentially omitting the single studies and recounting the pooled ORs and 95% CIs was performed to estimate the effect of individual studies on overall risk of cancer. The funnel plot was utilized to test the publication bias and Egger’s test (linear regression analysis) was used to check the symmetry of funnel plots^18^.

STATA software (version 12.0, Stata Corporation, College Station, TX) was performed to analyze statistical data. All tests were two-tailed and the significance level was fixed at .10.

## Results

### Selection of studies

**Figure 1:**
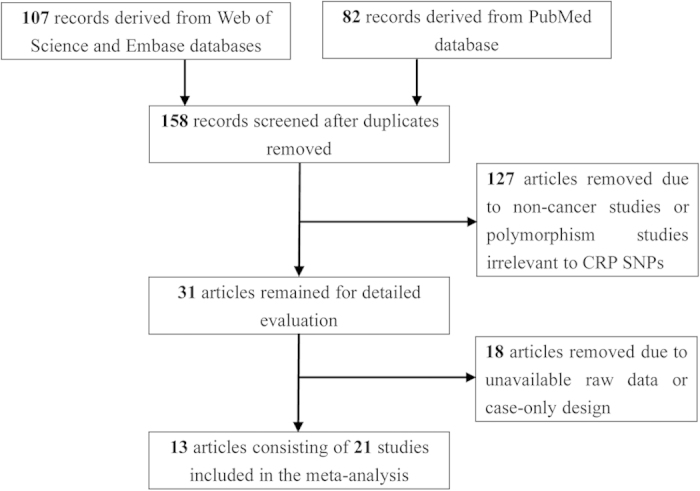
Flow diagram of the study selection process.

As showed in Fig. 1, we derived 107 records from Web of Science and Embase, and 82 records from PubMed. We screened all 189 records and first excluded 31 duplicates. After title and abstract evaluation, 127 articles were removed due to non-cancer studies or polymorphism studies irrelevant to *CRP* SNPs. Of the 31 remaining articles, 24 were eventually excluded because of unavailable raw data or case-only design. As a result, 7 articles consisting of 7 case-control studies of +942G>C polymorphism and 7 studies of 1846C>T polymorphism were considered in the final analysis^13^,^14^,^15^,^19^,^20^,^21^,^22^.

### Characteristics of studies

Summary characteristics of the studies included are described in Table 2. There were 7 studies for +942G>C polymorphism, of which 4 studies used Caucasians and 3 studies used East Asians. Four types of cancer were investigated, including colorectal cancer, lung cancer, esophageal cancer and endometrial cancer. Since two studies only reported allele frequency or genetic data for GG, GC + CC^13^,^21^, these data were analyzed in the allele model or the dominant model. In terms of 1846C>T polymorphism, the pooling dataset was composed of 4 colorectal cancer studies, 2 lung cancer studies and 1 study of esophageal cancer. In addition, both Caucasian and Asian ethnicities were studied. All studies were in HWE with the exception of a Caucasian study reporting on the connection between colorectal cancer and 1846C>T polymorphism^15^.

### Quantitative synthesis

#### Association between cancer risk and +942G>C polymorphism

**Figure 2:**
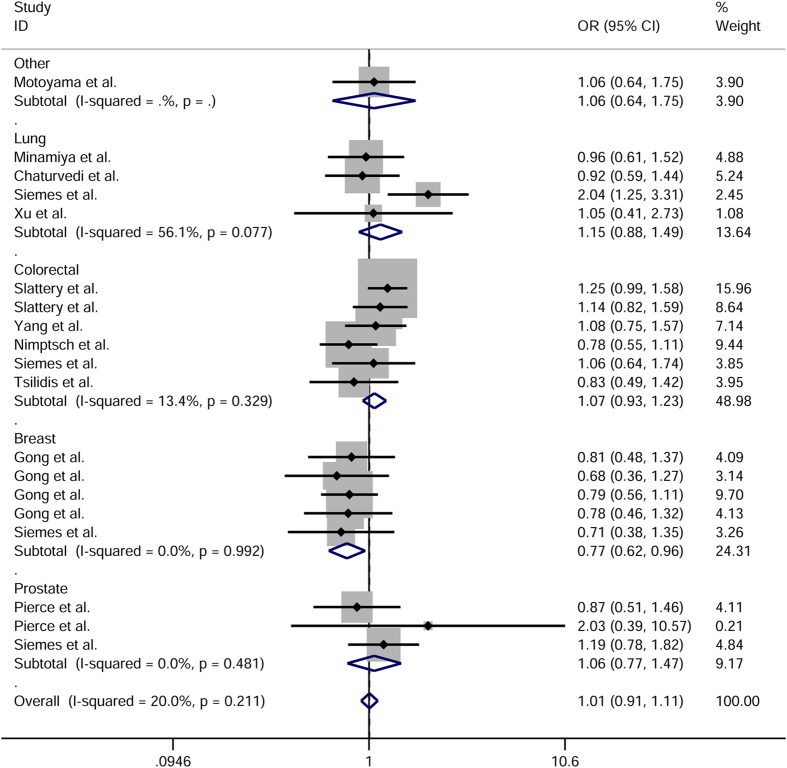
Forest plot of cancer susceptibility in relation to *CRP* +942G>C polymorphism in CC vs. GG model.

As shown in Fig. 2, meta-analysis of 4 450 cancer cases and 5 165 controls demonstrated that the CC genotype was significantly associated with 3.71 fold increased risk of overall cancer compared to the GG genotype (CC vs. GG: OR = 3.71, 95% CI = 1.56–8.79, *P*_*heterogeneity*_ = 0.590). The CC vs. GC + GG genetic model also provided an OR of 3.79 (95% CI = 1.60–8.98, *P*_*heterogeneity*_ = 0.571, Table 1), suggesting individuals with the CC genotype had almost 4 fold higher risk of cancer than those with the GC and GG genotypes.

We then performed stratified analysis by cancer type and found an almost 5 fold greater risk of colorectal cancer associated with the CC genotype of +942G>C polymorphism (CC vs. GG: OR = 4.43, 95% CI = 1.63–12.08, *P*_*heterogeneity*_ = 0.501; CC vs. GC + GG: OR = 4.56, 95% CI = 1.68–12.41, *P*_*heterogeneity*_ = 0.487).

#### Association between cancer risk and 1846C>T polymorphism

**Figure 3:**
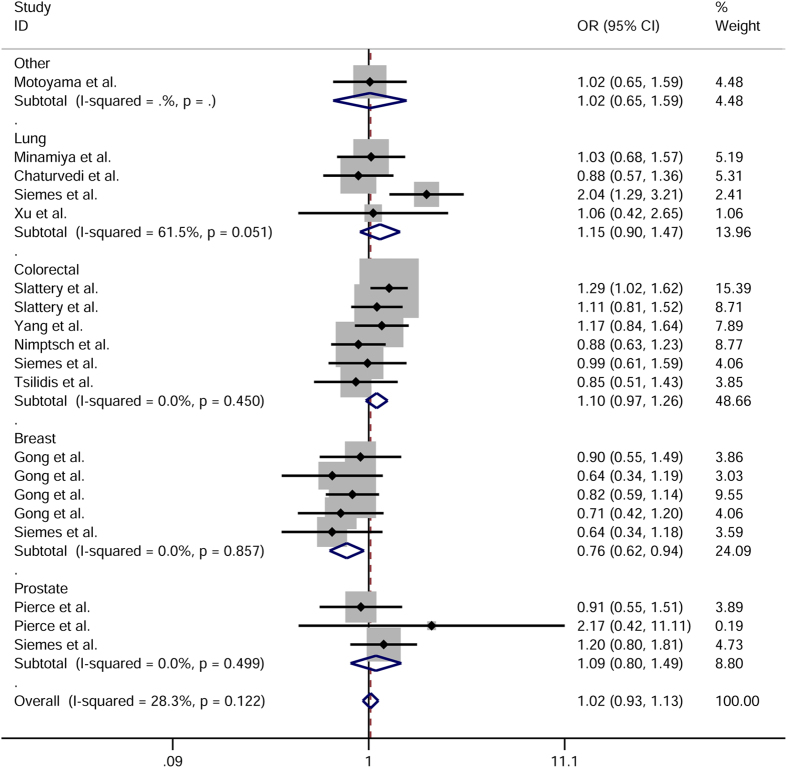
Forest plot of cancer susceptibility in relation to *CRP* 1846C>T polymorphism in TT vs. CC model.

A total of 3 543 cases and 4 263 controls were analyzed in this meta-analysis. On the whole, the 1846 TT genotype was found to increase cancer risk when all case-control studies were pooled (TT vs. CC: OR = 1.15, 95% CI = 1.01–1.31, *P*_*heterogeneity*_ = 0.846, Fig. 3; TT vs. CT + CC; OR = 1.17, 95% CI = 1.03–1.32, *P*_*heterogeneity*_ = 0.656).

When we performed stratification analyses by ethnicity, significantly elevated risk of cancer was found in Caucasians using the TT vs. CC (OR = 1.19, 95% CI = 1.02–1.39, *P*_*heterogeneity*_ = 0.630) and TT vs. CT + CC genetic model (OR = 1.21, 95% CI = 1.04–1.40, *P*_*heterogeneity*_ = 0.355). The same two genetic models showed an association with colorectal cancer when analysis was constrained to cancer type (Table 1).

### Sensitivity analysis

To evaluate the influence of individual studies on risk of overall cancer, we performed leave-one-out sensitivity analysis and recomputed the pooled ORs. The ORs calculated after excluding a single study did not show any differences from the primary values. This process assured the stability of overall results (Figure not shown).

### Publication bias

**Figure 4:**
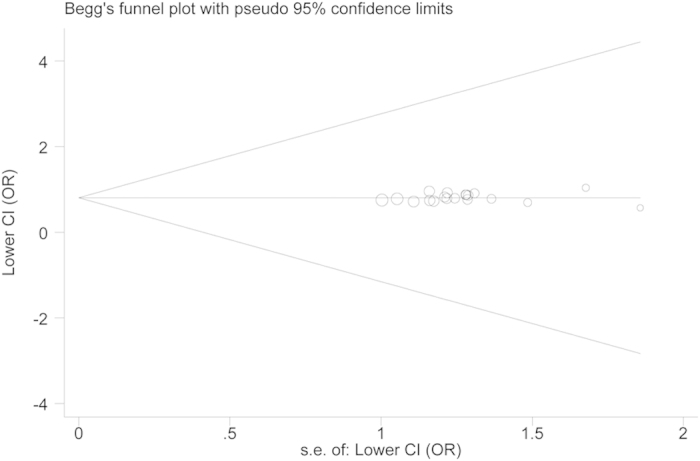
Funnel plots of *CRP* +942G>C polymorphism and cancer susceptibility.

**Figure 5:**
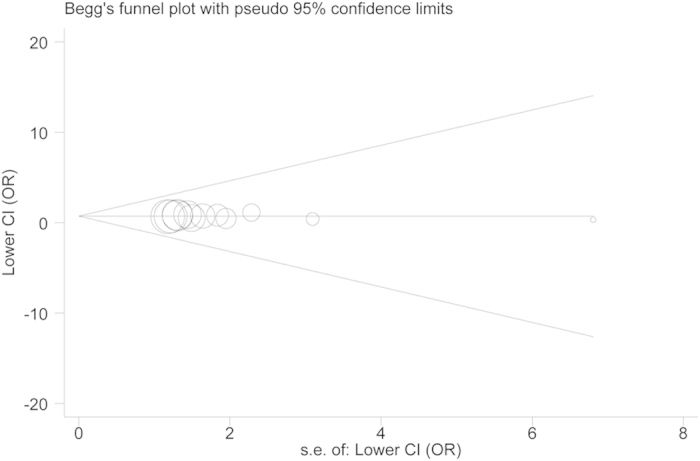
Funnel plots of *CRP* 1846C>T polymorphism and cancer susceptibility.

Evaluation of publication bias was performed using both Begg’s funnel plot and Egger’s test. The studies of *CRP* polymorphisms were symmetrically distributed (Fig. 4, +942G>C, C vs. G; Fig. 5, 1846C>T, T vs. C), which was confirmed by the Egger’s test (*P* = 0.352; *P* = 0.628). Therefore, our meta-analysis results are not affected by publication bias and worthy of trust.

## Discussion

To the best of our knowledge, this is the first quantitative assessment of the genetic association studies reporting on the relationship between *CRP* polymorphisms and cancer susceptibility. This meta-analysis summarized a total of 8 case-control studies, providing evidence that supported a significant role of *CRP* polymorphisms in cancer. More specifically, the CC genotype of +942G>C polymorphism was associated with significantly increased risk of cancer, particularly colorectal cancer. We also noted that the carriage of 1846 TT genotype had higher risk to develop cancer. Subgroup analysis by ethnicity and cancer type showed a similar trend towards an increased risk in Caucasians and colorectal cancer. These data suggest that genetic polymorphisms in the *CRP* gene may have effects on the development of cancer.

As inflammation is important in the progression of human cancer, much attention has been directed to the *CRP*, an inflammation-related gene. Several lines of work have connected the functional polymorphisms at *CRP* locus with cancer. For SNP +942G>C, Wen and workmates investigated the association with endometrial cancer in a relatively large study, suggesting *CRP* +942G>C alone was not associated with this cancer in Chinese patients^13^. Such an insignificant association was seen in most of the published studies representing distinct ethnicities^14^,^15^,^20^. These observations are in disagreement with those suggested in the current study, where increased susceptibility of cancer was revealed. As most individually published studies, even in different populations, shared the same finding that *CRP* +942G>C was not an independent risk factor for cancers, the most persuasive explanation to the discrepancy is the inadequate statistical power caused by the small number of subjects in these studies. In addition, it should be noted that *CRP* +942G>C itself may not modify cancer risk, but it influences cancer development by interacting with body mass index (BMI) and family history of cancer^15^, implicating the biological functions of *CRP* +942G>C are possibly determined by physical conditions of individuals themselves.

In terms of *CRP* 1846C>T, previous reports have generated controversial results. A nested case-control study of lung cancer in 1 262 samples of Caucasian descent suggested that the association between 1846C>T and lung cancer was not statistically significant^20^. In contrast, a Caucasian study demonstrated a decreased risk of colorectal cancer in relation to the C allele^21^, an observation contradicted a later colorectal cancer study in which the same allele was found to have a fixed 30% increased colorectal cancer risk^15^. Again, inadequacy of study sample may be responsible a large part for the existing inconsistency. An alternative explanation may relate to the differences in cancer type, because etiology of various human cancers is heterogeneous and complex, studies of a single polymorphism seem impossible to determine the association with cancer.

Our findings are supported by previous mechanic studies. The study by Heikkila and workmates suggested that elevated expression of *CRP* was linked to increased overall risk of cancer (RR = 1.10, 95% CI = 1.02–1.18); the association was more pronounced in lung cancer (RR = 1.32, 95% CI = 1.08–1.61)^23^. Likewise, a subsequent meta-analysis analyzed 1 918 lung cancer cases and revealed significantly increased risk of lung cancer associated with higher *CRP* levels among men (RR = 1.18, 95% CI = 1.09–1.28)^24^. According to epidemiologic data, the increased *CRP* levels should be attributable to the genetic variations in the *CRP* gene^25^, including +942G>C and 1846C>T polymorphisms^26^,^27^. Therefore, it is plausible that *CRP* polymorphisms correlate with cancer development. For the significant association observed in Caucasians, but not in Asians, there are several possibilities, one of which relates to the remarkably different expression patterns of *CRP* in non-homogeneous ethnic populations^25^.

Our findings should be interpreted with caution due to a few potential limitations. First, we may have missed some studies containing usable data, even though an exhaustive literature search was undertaken in the PubMed, a database we put more emphasis on. To maximize the case panel in this analysis, we additionally searched Web of Science and Embase in which a large number of medical research papers are collected. Second, selection bias may have been introduced, as we included both population-based studies randomly selecting controls from individuals with no lesions and hospital-based studies using healthy controls ascertained through routine health check. Third, despite a significant association with overall risk of cancer and colorectal cancer was suggested in this meta-analysis, we only can infer but cannot conclude that *CRP* polymorphisms are susceptibility loci of other types of cancer, highlighting the necessity for further investigation.

In conclusion, we found that genetic polymorphisms in the *CRP* gene, +942G>C and 1846C>T, are associated with an increased overall risk of cancer. Subgroup analyses by ethnicity and cancer type also showed an significant association in Caucasians and colorectal cancer. Future research is quite necessary to provide compelling evidence of the association between *CRP* gene polymorphisms and cancer risk.

## Additional Information

**How to cite this article**: Geng, P. *et al*. Genetic polymorphisms in C-reactive protein increase cancer susceptibility. *Sci. Rep*. **5**, 17161; doi: 10.1038/srep17161 (2015).

## Figures and Tables

**Table 1:** Quantitative analyses of *CRP* polymorphism on cancer risk.

Variables	N†	Heterozygous		Homozygous		Allele		Dominant		Recessive	
		OR (95% CI)	*P*‡	OR (95% CI)	*P*‡	OR (95% CI)	*P*‡	OR (95% CI)	*P*‡	OR (95% CI)	*P*‡
Total	4450,5165	0.94 (0.81, 1.10)	0.745	3.71 (1.56, 8.79)	0.590	1.03 (0.90,1.17)	0.859	1.00 (0.86, 1.15)	0.933	3.79 (1.60, 8.98)	0.571
Cancer type											
CRC	2916,3544	0.93 (0.78, 1.09)	0.437	4.43 (1.63, 12.08)	0.501	1.03 (0.88,1.20)	0.409	0.99 (0.85, 1.15)	0.756	4.56 (1.68,12.41)	0.487
Others	1534,1621	1.04 (0.69, 1.57)	0.905	1.78 (0.30,10.71)	–	1.03 (0.82,1.28)	0.934	1.07 (0.71, 1.59)	0.941	1.77 (0.29,10.67)	–
1846C>T		CT vs. CC		TT vs. CC		T vs. C		TT + CT vs. CC		TT vs. CT + CC	
Total	3543,4263	1.02 (0.94, 1.11)	0.993	1.15 (1.01, 1.31)	0.846	1.06 (0.99,1.12)	0.934	1.03 (0.96, 1.11)	0.997	1.17 (1.03, 1.32)	0.656
Ethnicity											
Caucasian	2866,3767	1.02 (0.93, 1.12)	0.912	1.19 (1.02, 1.39)	0.630	1.06 (0.99,1.14)	0.675	1.04 (0.95, 1.12)	0.940	1.21 (1.04, 1.40)	0.355
Asian	677,496	1.00 (0.79, 1.25)	0.913	1.04 (0.81, 1.34)	0.924	1.03 (0.90,1.18)	0.919	1.01 (0.85, 1.20)	0.960	1.09 (0.87, 1.37)	0.834
Cancer type											
CRC	2912,3539	1.02 (0.93, 1.11)	0.916	1.21 (1.04, 1.40)	0.871	1.07 (1.00,1.15)	0.787	1.03 (0.95, 1.12)	0.938	1.24 (1.08, 1.43)	0.785
Others	631,724	1.03 (0.84, 1.26)	0.889	0.98 (0.75, 1.28)	0.917	1.00 (0.87,1.15)	0.975	1.01 (0.85, 1.20)	0.956	0.97 (0.76, 1.25)	0.857

CRC-colorectal cancer, ^†^-number of cases and controls, ^‡^-p value for heterogeneity test.

**Table 2:** Main characteristics of all seven studies included in a meta-analysis.

Authors	Year	Country oforigin	Ethnicity	Cancer type	Polymorphismstudied	Genotypedcases	Genotypedcontrols
Wen *et al*.	2008	China	East Asian	Endometrial cancer	+942G>C	1046	1035
Motoyama *et al*.	2009	Japan	East Asian	Esophageal cancer	+942G>C, 1846C>T	110, 110	139,139
Minamiya *et al*.	2010	Japan	East Asian	Lung cancer	1846C>T	146	139
Chaturvedi *et al*.	2010	USA	Caucasian	Lung cancer	+942G>C, 1846C>T	378,375	447,446
Ognjanovic *et al*.	2010	USA	Caucasian	Colorectal cancer	+942G>C, 1846C>T	268,270	536,536
Slattery *et al*.	2011	USA	Caucasian	Colorectal cancer	+942G>C, 1846C>T	1523,1522	1887,1884
Slattery *et al*.	2011	USA	Caucasian	Colorectal cancer	+942G>C, 1846C>T	705,699	903,901
Yang *et al*.	2011	China	East Asian	Colorectal cancer	+942G>C, 1846C>T	420,421	218,218
